# Tadalafil treatment in mice for preeclampsia with fetal growth restriction has neuro-benefic effects in offspring through modulating prenatal hypoxic conditions

**DOI:** 10.1038/s41598-018-36084-x

**Published:** 2019-01-18

**Authors:** Ryota Tachibana, Takashi Umekawa, Kento Yoshikawa, Takao Owa, Shoichi Magawa, Fumi Furuhashi, Makoto Tsuji, Shintaro Maki, Kyoko Shimada, Michiko K. Kaneda, Masafumi Nii, Hiroaki Tanaka, Kayo Tanaka, Yuki Kamimoto, Eiji Kondo, Ineko Kato, Kenji Ikemura, Masahiro Okuda, Ning Ma, Takekazu Miyoshi, Hiroshi Hosoda, Masayuki Endoh, Tadashi Kimura, Tomoaki Ikeda

**Affiliations:** 1 Department of Obstetrics and Gynecology, Mie University Graduate School of Medicine, Tsu, Japan; 2 Department of Obstetrics and Gynecology, Osaka University Graduate School of Medicine, Osaka, Japan; 3 Department of Pharmacy, Mie University Hospital, Tsu, Japan; 4 Faculty of Health Science, Suzuka University of Medical Science, Suzuka, Japan; 5 Department of Perinatology and Gynecology, National Cerebral and Cardiovascular Center, Suita, Japan; 6 Department of Regenerative Medicine and Tissue Engineering, National Cerebral and Cardiovascular Center, Suita, Japan; 7 Clinical Research Support Center, Mie University Hospital, Tsu, Japan

## Abstract

We have demonstrated that tadalafil facilitates fetal growth in mice with L-NG-nitroarginine methyl ester (L-NAME)-induced preeclampsia (PE) with fetal growth restriction (FGR). Tadalafil is a selective phosphodiesterase 5 inhibitor that dilates the maternal blood sinuses in the placenta, thereby facilitating the growth of the fetus. The purpose of this study was to investigate the effects of tadalafil treatment for PE and FGR on the developing brain in FGR offspring using an L-NAME-induced mouse model of PE with FGR. A control group of dams received carboxymethylcellulose (CMC). L-NAME-treated groups received L-NAME dissolved in CMC from 11 days post coitum (d.p.c.). The L-NAME-treated dams were divided into two subgroups 14 d.p.c. One subgroup continued to receive L-NAME. The other subgroup received L-NAME with tadalafil suspended in CMC. Tadalafil treatment for PE with FGR reduced the expression of hypoxia-inducible factor-2α in the placenta and in the brain of the FGR fetus. Moreover, tadalafil treatment *in utero* shows improved synaptogenesis and myelination in FGR offspring on postnatal day 15 (P15) and P30. These results suggest that tadalafil treatment for PE with FGR not only facilitates fetal growth, but also has neuroprotective effects on the developing brain of FGR offspring through modulating prenatal hypoxic conditions.

## Introduction

Fetal growth restriction (FGR), in which the fetus has failed to achieve genetic growth potential *in utero*^1^, is associated with an increased risk of motor and sensory neurodevelopmental deficits, and cognitive and learning impairments^2^. Preeclampsia (PE) is often associated with FGR, since both PE and FGR are disorders rooted in defects in early placental development^3^. The failure of trophoblast invasion and remodeling of the uterine spiral arteries leads to high resistance in uterine circulation, causing a reduction in blood flow and placental ischemia. Getahun *et al*. demonstrated that PE is independently associated with an increased risk of attention deficit/hyperactivity disorder (ADHD) in a large population-based study and suggested that *in utero* exposure to chronic ischemic-hypoxic conditions is associated with ADHD in childhood^4^.

Nitric oxide (NO) is produced by NO synthases and regulates vascular tone in the placenta. Placental blood vessels express molecular mediators of the NO-dependent pathway, including a cyclic guanosine monophosphate (cGMP)-specific phosphodiesterase (PDE)^5^. Because inhibitors of PDE5, which is a cGMP-specific PDE, exert their pharmacological action by dilating arteries and increasing blood flow in erectile dysfunction and pulmonary hypertension^6^, recent studies have suggested a potential therapeutic role for PDE5 inhibitors in treating PE and FGR^7,8^. Tadalafil, a selective PDE5 inhibitor, has been used to treat pulmonary hypertension in pregnant women. We have recently shown a potential therapeutic effect for tadalafil on PE and FGR in small clinical trials^9–12^. In addition, our animal experiments have demonstrated that tadalafil treatment elevates maternal urinary excretion of cGMP and dilates the maternal blood sinuses in the placenta, which facilitates fetal growth^13^. These findings led us to hypothesize that tadalafil treatment for PE and FGR may improve neurodevelopment in offspring. The aim of this study was to investigate the effects of 5tadalafil treatment for PE and FGR on the developing brain in offspring using an L-NG-nitroarginine methyl ester (L-NAME)-induced mouse model of PE with FGR.

## Results

### Study 1: Effect of tadalafil treatment for PE with FGR on the fetal brain

#### Effect of tadalafil treatment for PE with FGR on maternal parameters

We used the PE with FGR mouse model induced by L-NAME as described previously, with a small modification^13^. A control group of dams (C dam, n = 5) received 0.5% carboxymethylcellulose (CMC). L-NAME-treated groups received 1 mg/ml L-NAME dissolved in CMC from 11 days postcoitum (d.p.c.). The L-NAME-treated dams were divided into two subgroups 14 d.p.c. One subgroup continued to receive L-NAME until 17 d.p.c. (L dam, n = 8). The other subgroup received L-NAME with 0.08 mg/ml tadalafil suspended in CMC until 17 d.p.c. (TL dam, n = 5). The maternal body weight (BW), the maternal weight gain from 14 to 17 d.p.c., and the maternal daily food intake did not differ significantly among the 3 groups (Supplementary Table S1). The average dose of L-NAME was 220.0 ± 16.9 mg/kg BW/day. The average dose of tadalafil was 13.9 ± 1.9 mg/kg BW/day. The mean systolic blood pressure (SBP) measured by tail-cuff methods was significantly higher for the L and the TL dams compared to the C dams 14 d.p.c. (C dams, 106.8 ± 3.0 mm Hg; L dams, 126.7 ± 5.1 mm Hg; TL dams, 126.7 ± 7.0 mm Hg; Supplementary Fig. S1A). The L dams had a significantly higher mean SBP than the C dams, but the mean SBP improved significantly for the TL dams in comparison with the L dams 16 d.p.c. (C dams, 101.3 ± 7.8 mm Hg; L dams, 126.5 ± 3.3 mm Hg; TL dams, 113.1 ± 4.7 mm Hg; Supplementary Fig. S1B).

#### Effect of tadalafil treatment for PE with FGR on the fetal brain

**Figure 1:**
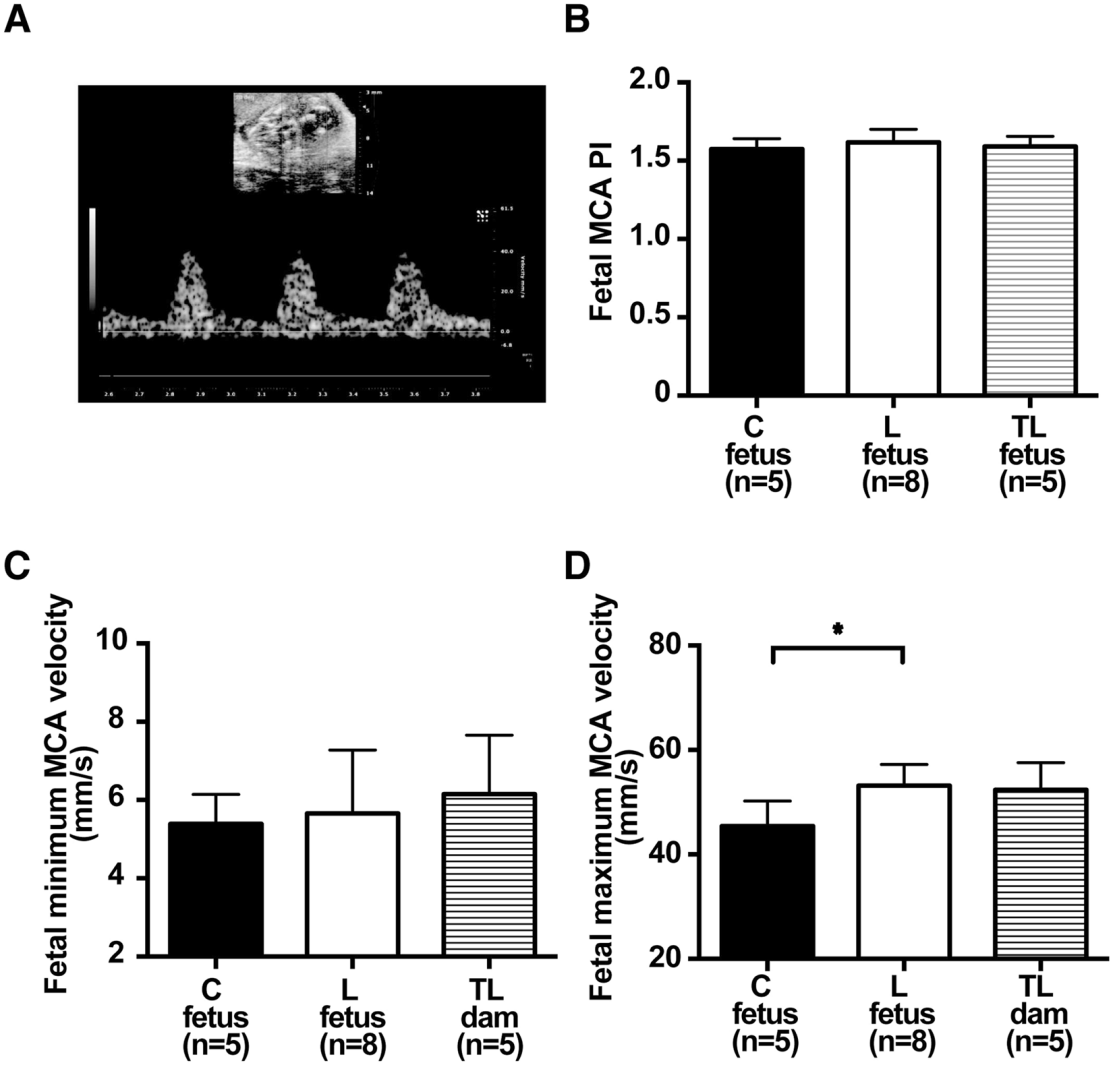
Fetal middle cerebral artery (MCA) flow 17 d.p.c. (**A**) Representative Doppler waveforms of fetal MCA. (**B**) Mean fetal MCA pulsatility index (PI) 17 d.p.c. for C fetus (n = 5), L fetus (n = 8), and TL fetus (n = 5). (**C**) Mean minimum MCA velocity 17 d.p.c. for C fetus (n = 5), L fetus (n = 8), and TL fetus (n = 5). (**D**) Mean maximum MCA velocity 17 d.p.c. for C fetus (n = 5), L fetus (n = 8), and TL fetus (n = 5). Values are presented as mean ± SD. Asterisks show statistically significant differences (*P* < 0.05) between groups indicated by square brackets as determined by one-way ANOVA followed by Tukey’s post-hoc test. C fetus: fetus from C dam. L fetus: fetus from L dam. TL fetus: fetus from TL dam.

We investigated fetal middle cerebral artery (MCA) flow velocity with Doppler ultrasound biomicroscopy 17 d.p.c. (Fig. 1A). There was no difference in the fetal MCA pulsatility index (PI) among the 3 groups (C fetus, 1.57 ± 0.07; L fetus, 1.61 ± 0.08; TL fetus, 1.59 ± 0.07; *P* = 0.58; Fig. 1B). Fetal minimum MCA velocity did not differ significantly among the 3 groups (C fetus, 5.4 ± 0.8 mm/s; L fetus, 5.7 ± 1.6 mm/s; TL fetus, 6.2 ± 1.5 mm/s; *P* = 0.69; Fig. 1C). However, notably, fetal maximum MCA velocity was significantly higher for the L dams than the C dams, whereas there were no significant differences between that for the C dams and the TL dams (C fetus, 45.4 ± 4.8 mm/s; L fetus, 53.2 ± 4.1 mm/s; TL fetus, 52.4 ± 5.2 mm/s; Fig. 1D). Fetal BW 17 d.p.c. was significantly lower for L dams than C dams. In contrast, fetal BW was improved significantly for TL dams compared to L dams (C fetus, 0.85 ± 0.05 g; L fetus, 0.75 ± 0.08 g; TL fetus, 0.80 ± 0.06 g; Supplementary Fig. S2). Placental weight was significantly lower for both the L dams and the TL dams than for the C dams (C placenta, 0.088 ± 0.010 g; L placenta, 0.082 ± 0.010 g; TL placenta, 0.081 ± 0.010 g; Supplementary Fig. S2). The concentrations of tadalafil in the placenta and in the fetal brain were 5.9 ± 0.5 ng/mg protein (n = 4) and 4.5 ± 2.0 ng/mg protein (n = 3), respectively.

**Figure 2:**
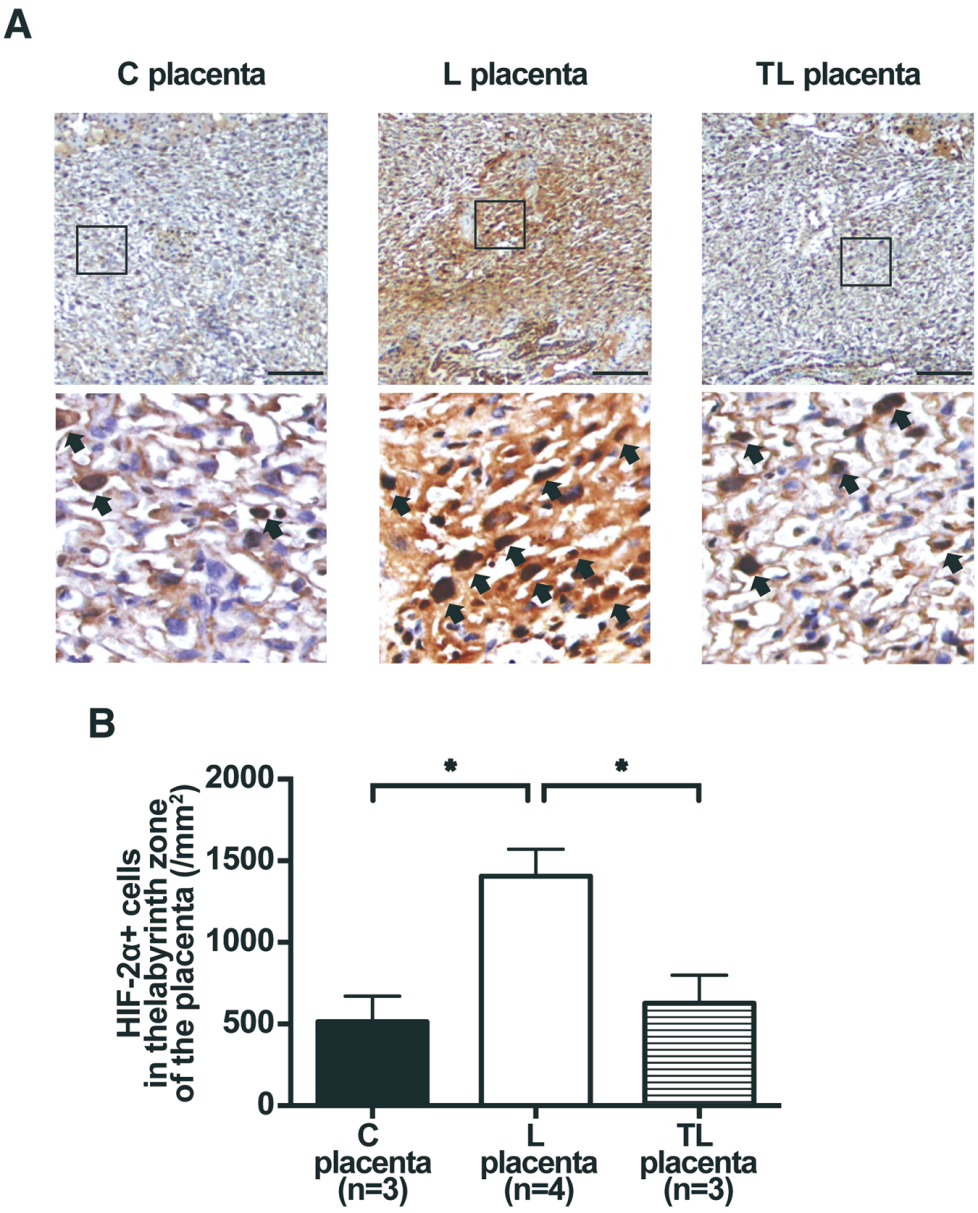
HIF-2α expression in the labyrinth zone of the placenta 17 d.p.c. (**A**) Representative images of HIF-2α expression (arrows) in the labyrinth zone of the (**C**) placenta, the L placenta, and the TL placenta 17 d.p.c. Scale bars: 100 μm. The areas in the small rectangles are shown at higher magnification in the row below. Nuclei were counterstained with hematoxylin. (**B**) Number of HIF-2α positive (HIF-2α + ) cells in the labyrinth zone of the placenta 17 d.p.c. for C placenta (n = 3), L placenta (n = 4), and TL placenta (n = 3). Values are presented as mean ± SD. Asterisks show statistically significant differences (*P* < 0.05) between groups indicated by square brackets as determined by one-way ANOVA followed by Tukey’s post-hoc test. (**C**) placenta: placenta from C dam. L placenta: placenta from L dam. TL placenta: placenta from TL dam.

**Figure 3:**
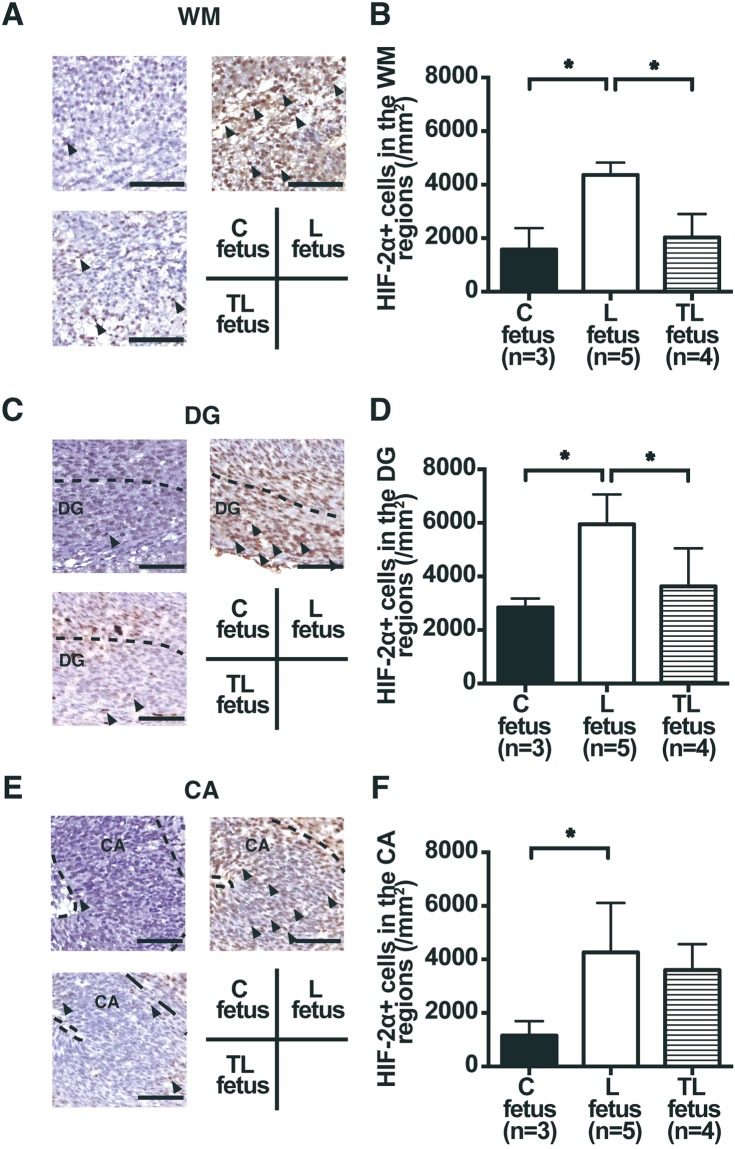
HIF-2α expression in the white matter (WM) and in the hippocampus of the FGR fetal brain 17 d.p.c. (**A**,**C**,**E**) Representative images of HIF-2α expression (arrows) in the white matter (WM) regions (A), the dentate gyrus (DG) regions of the fetal hippocampus (shown by the broken line; **C**), and the cornu ammonis (CA) regions of the fetal hippocampus (shown by the broken line; **E**) 17 d.p.c. for C fetus, L fetus, and TL fetus. Scale bars: 50 μm. Nuclei were counterstained with hematoxylin. (**B**,**D**,**F**) Number of HIF-2α positive (HIF-2α+) cells in the WM regions (**B**), in the DG regions (**D**), and the CA regions (**F**) of the fetal hippocampus 17 d.p.c. for C fetus (n = 3), L fetus (n = 5), and TL fetus (n = 4). Values are presented as mean ± SD. Asterisks show statistically significant differences (*P* < 0.05) between groups indicated by square brackets as determined by one-way ANOVA followed by Tukey’s post-hoc test. C fetus: fetus from C dam. L fetus: fetus from L dam. TL fetus: fetus from TL dam.

The hypoxic conditions in the placenta and in the fetal brain were assessed by the expression of hypoxia-inducible factor (HIF)-2α. The number of HIF-2α positive (HIF-2α+) cells in the labyrinth zone of the placenta was higher for the L dams than for the C dams. In contrast, the number of HIF-2α + cells in the labyrinth zone of the placenta was decreased significantly for the TL dams compared to the L dams (C placenta, 513.9 ± 157.0/mm^2^; L placenta, 1406.3 ± 164.8/mm^2^; TL placenta, 627.3 ± 172.1/mm^2^; Fig. 2). The number of HIF-2α + cells in the white matter (WM) regions (C fetus, 1577.8 ± 800.9/mm^2^; L fetus, 4360.0 ± 460.9/mm^2^; TL fetus, 2033.3 ± 868.4/mm^2^; Fig. 3A) and in the dentate gyrus (DG) regions of the fetal hippocampus (C fetus, 2844.4 ± 335.5/mm^2^; L fetus, 5946.7 ± 1114.0/mm^2^; TL fetus, 3633.3 ± 1416.8/mm^2^; Fig. 3B) were higher for the L dams than for the C dams, but the number of HIF-2α + cells in the WM regions and the DG regions was reduced significantly for the TL dams compared to the L dams. The number of HIF-2α + cells in the cornu ammonis (CA) regions of the fetal hippocampus was higher for the L dams than for the C dams, whereas there were no significant differences between that for the C dams and the TL dams (C fetus, 1155.6 ± 538.9/mm^2^; L fetus, 4266.7 ± 1845.1/mm^2^; TL fetus, 3600.0 ± 967.6/mm^2^; Fig. 3C).

### Study 2 Effect of tadalafil treatment for PE with FGR on the developing brain of FGR offspring on P15 and P30

Next, another set of dams (C dams, n = 4; L dams, n = 5; TL dams, n = 6) was prepared as described above in Study 1, and the dams were allowed to deliver spontaneously. All dams were given normal drinking water during lactation. Only male pups were used in this study to minimize the possible influence of sexual dimorphism.

### Effect of tadalafil treatment for PE with FGR on glial fibrillary acidic protein (GFAP) expression in the corpus callosum (CC) of FGR offspring on P15 and P30

**Figure 4:**
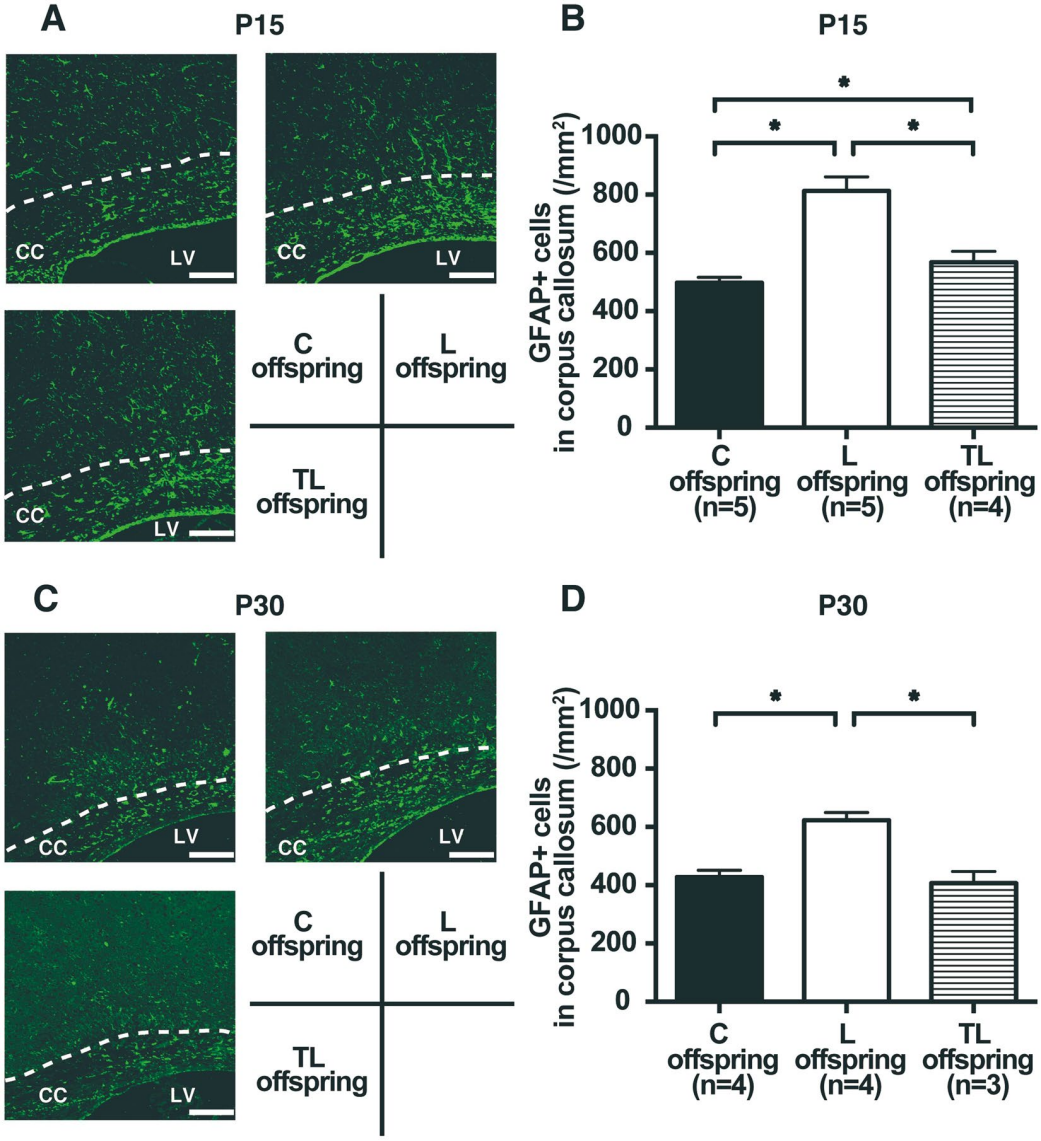
Expression of GFAP in the corpus callosum (CC) of FGR offspring on P15 and P30. (**A**,**C**) Representative images of GFAP expression (green) in the CC (shown by the broken line) of FGR offspring on P15 (**A**) and P30 (**C**) for C offspring, L offspring, and TL offspring. CC: corpus callosum. LV: lateral ventricle. Scale bars: 100 μm. (**B**,**D**) The number of GFAP positive (GFAP+) cells in the CC of FGR offspring on P15 (C offspring (n = 5), L offspring (n = 5), and TL offspring (n = 4)); (**B**) and P30 (C offspring (n = 4), L offspring (n = 4), and TL offspring (n = 3)); (**D**) Values are presented as mean ± SD. Asterisks show statistically significant differences (*P* < 0.05) between groups indicated by square brackets as determined by one-way ANOVA followed by Tukey’s post-hoc test. (C) offspring: fetus from C dam. L offspring: fetus from L dam. TL offspring: fetus from TL dam.

To examine the effect of tadalafil treatment for PE with FGR on astrogliosis in the brains of offspring, sections through the CC on postnatal day 15 (P15) and P30 were labeled for GFAP by fluorescent immunohistochemistry (Fig. 4A,C). The number of GFAP positive (GFAP+) cells in the CC of FGR offspring on P15 (C offspring, 498.3 ± 18.2/mm^2^; L offspring, 812.9 ± 48.1/mm^2^; TL offspring, 567.9 ± 37.6/mm^2^; Fig. 4B) and P30 (C offspring, 428.0 ± 22.9/mm^2^; L offspring, 623.3 ± 26.3/mm^2^; TL offspring, 407.0 ± 40.2/mm^2^; Fig. 4D) was higher for the L dams than for the C dams, whereas it was decreased significantly for the TL offspring compared to the L offspring. The number of GFAP + cells in the CC was higher for the TL offspring than the C offspring on P15, whereas there were no significant differences between the C and the TL offspring on P30 (Fig. 4B,D).

### Effect of tadalafil treatment on myelin basic protein (MBP) expression in the cingulum of FGR offspring on P15 and P30

**Figure 5:**
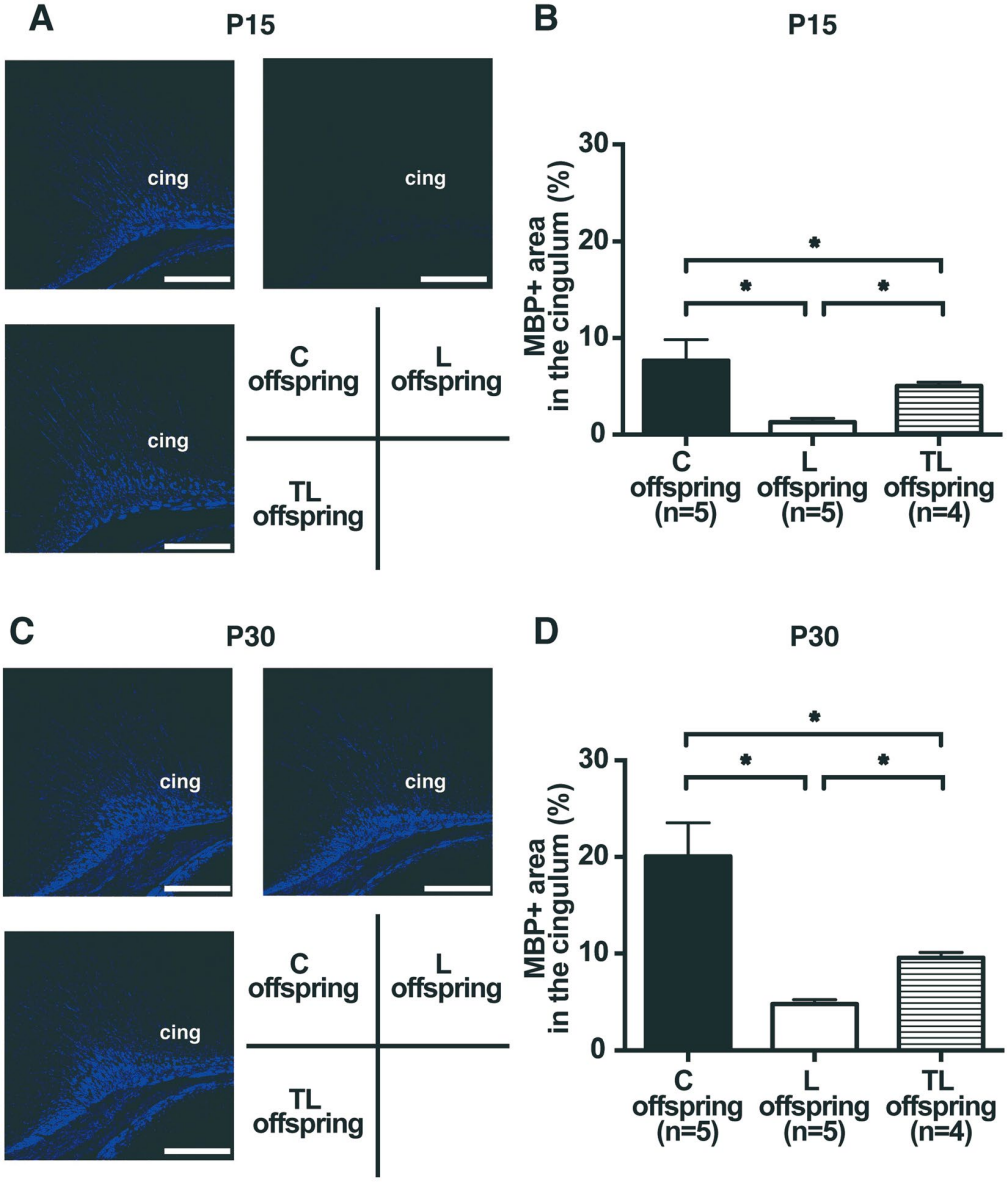
Expression of MBP in the cingulum of FGR offspring on P15 and P30. (**A**,**C**) Representative images of MBP expression (blue) in the cingulum of FGR offspring on P15 (**A**) and P30 (**C**) for C offspring, L offspring, and TL offspring. cing: cingulum. Scale bars: 200 μm. (**B**,**D**) The number of MBP positive (MBP+) cells in the cingulum of FGR offspring on P15 (C offspring (n = 5), L offspring (n = 5), and TL offspring (n = 4)); (**B**) and P30 (C offspring (n = 4), L offspring (n = 4), and TL offspring (n = 3)); (**D**) Values are presented as mean ± SD. Asterisks show statistically significant differences (*P* < 0.05) between groups indicated by square brackets as determined by one-way ANOVA followed by Tukey’s post-hoc test. C offspring: fetus from C dam. L offspring: fetus from L dam. TL offspring: fetus from TL dam.

We investigated the effect of tadalafil treatment for PE with FGR on myelination in the cingulum of FGR offspring on P15 and P30 (Fig. 5A,C). The MBP positive (MBP+) area in the cingulum on P15 (C offspring, 7.7 ± 2.2%; L offspring, 1.3 ± 0.4%; TL offspring, 5.0 ± 0.4%; Fig. 5B) and P30 (C offspring, 20.1 ± 3.5%; L offspring, 4.8 ± 0.5%; TL offspring, 9.6 ± 0.5%; Fig. 5D) was lower for the L dams than for the C dams, but was improved significantly for the TL offspring compared to the L offspring. The MBP + area in the hippocampus for the TL offspring was lower than for the C offspring on P15 and P30 (Fig. 5C,D).

### Effect of tadalafil treatment on synaptophysin expression in the hippocampus of FGR offspring on P15 and P30

**Figure 6:**
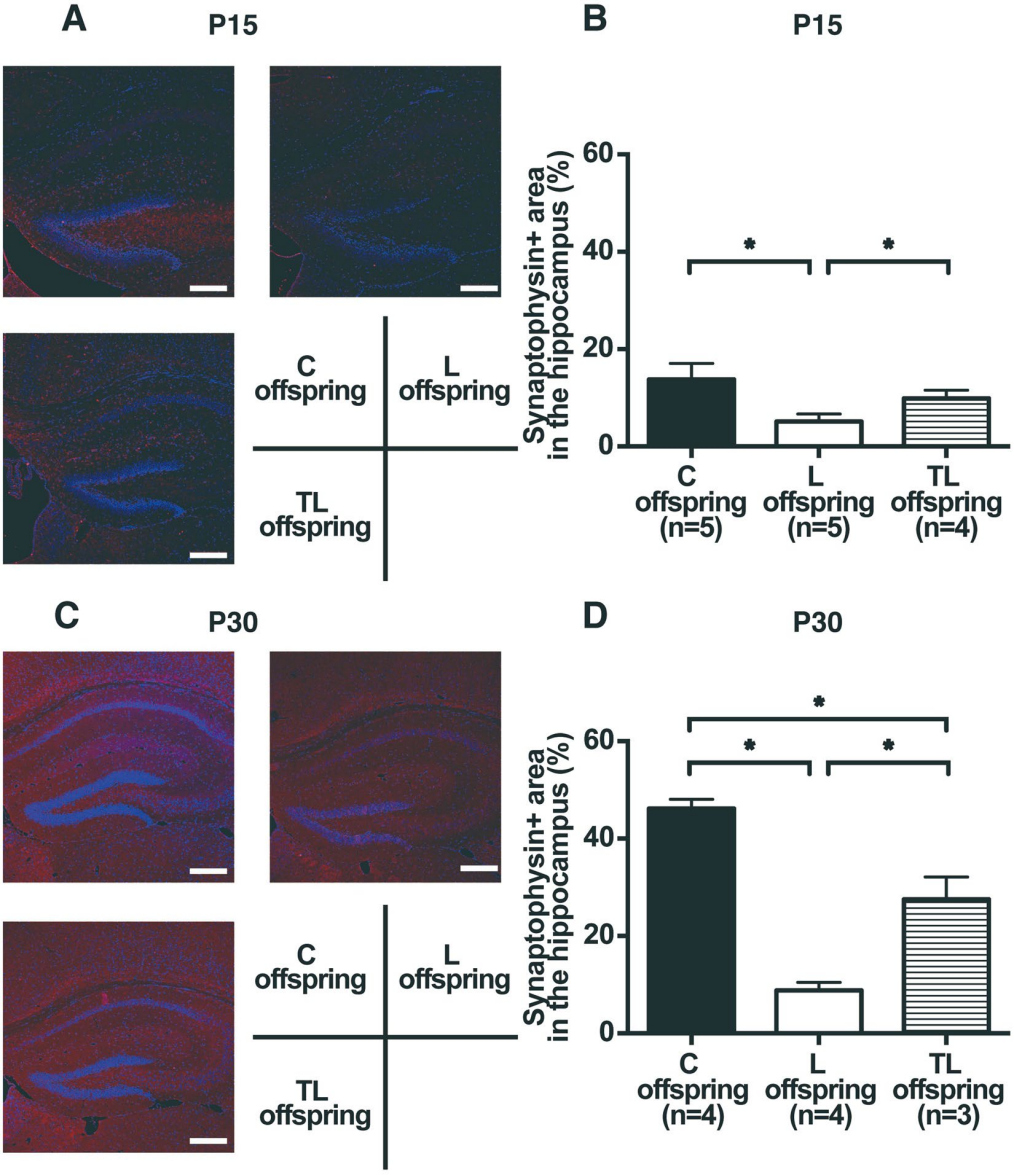
Expression of synaptophysin in the hippocampus of FGR offspring on P15 and P30. (**A**,**C**) Representative images of synaptophysin expression (red) in the hippocampus of FGR offspring on P15 (**A**) and P30 (**C**) for C offspring, L offspring, and TL offspring. Nuclei DAPI staining is shown in blue. Scale bars: 200 μm. (**B**,**D**) The number of synaptophysin positive (synaptophysin+) cells in the hippocampus of FGR offspring on P15 (C offspring (n = 5), L offspring (n = 5), and TL offspring (n = 4)); (**B**) and P30 (C offspring (n = 4), L offspring (n = 4), and TL offspring (n = 3)); (**D**). Values are presented as mean ± SD. Asterisks show statistically significant differences (*P* < 0.05) between groups indicated by square brackets as determined by one-way ANOVA followed by Tukey’s post-hoc test. C offspring: fetus from C dam. L offspring: fetus from L dam. TL offspring: fetus from TL dam.

Finally, to investigate the effect of tadalafil treatment for PE with FGR on hippocampal synaptogenesis in FGR offspring, we analyzed synaptophysin expression in the dorsal hippocampus on P15 and P30 (Fig. 6A,C). The synaptophysin positive (synaptophysin+) area in the hippocampus on P15 (C offspring, 13.8 ± 3.3%; L offspring, 5.1 ± 1.6%; TL offspring, 9.9 ± 1.7%; Fig. 6B) and P30 (C offspring, 46.2 ± 1.9%; L offspring, 8.8 ± 1.7%; TL offspring, 27.5 ± 4.6%; Fig. 6D) was more suppressed for the L dams than for the C dams. In contrast, the synaptophysin + area in the hippocampus was increased significantly for the TL offspring compared to the L offspring. There were no significant differences between the C and the TL offspring on P15, whereas the synaptophysin + area in the hippocampus for the TL offspring was lower than for the C offspring on P30 (Fig. 6B,D).

## Discussion

The main findings of the present study were, first, that tadalafil treatment for PE with FGR reduced the expression of HIF-2α in the placenta and in the brain of the FGR fetus. Second, neurodevelopment in FGR offspring in our mouse model was improved by tadalafil treatment *in utero*.

The defective trophoblast invasion seen with PE or FGR is thought to lead to relative hypoxia in the placenta^14^. HIF-1α and HIF-2α are central to the process of the adaptation to hypoxic conditions^15^. Recently, Fujii *et al*. have reported that HIF-2α expression, but not HIF-1α, in the placenta was increased and had a negative impact on placental growth factor (PlGF) production under hypoxic conditions^16^. HIF-2α expression was enhanced in the L-NAME-treated placenta and improved by tadalafil treatment in this study. Our previous report has demonstrated that tadalafil treatment during pregnancy leads to increased PlGF production in the placenta^13^. Trollmann *et al*. have reported that HIF-2α expression was elevated in the placenta and in the fetal brain after exposing pregnant mice to hypoxia^17^. In the other hands, HIF-1α was not different between C dams, L dams and TL dams. The reason for no change of HIF1α- is not clear in this study. HIF-2α is expressed in endothelial cells (ECs), neuronal cells, and other cells. Its activation in ECs correlates to vascular remodeling and inflammatory responses, whereas the expression of it in neurons indicates acute neuronal damage^17^. In the brain, HIF-2α is expressed in endothelial cells (ECs), neuronal cells, and other cells. Its activation in ECs correlates to vascular remodeling and inflammatory responses, whereas the expression of it in neurons indicates acute neuronal damage^18^. The effects of tadalafil may improve the inflammatory status in the FGR mice. Further studies are needed in order to evaluate effect on the inflammatory status of tadalafil.

Rosenberg *et al*. have shown that the maximum systolic velocity of the fetal cerebral arteries is better correlated with changes in brain blood flow during hypoxia than is the pulsatility index^19^. Thus, the results of the present study are supported by previous reports, and indicate that tadalafil treatment for PE with FGR reduces the influence of hypoxia on the fetal brain as well as on the placenta. Our previous data showed that the serum concentration of tadalafil in cord blood was approximately a quarter of that in the maternal blood^12^. Preeclampsia is caused contraction of the brain vessels. MCA maximum velocity may increase to measure the part of vasospastic narrowing. PI is affected by many factors in contrast to MCA maximum velocity, e.g. cardiac function, damage to the capillary endothelium. Therefore, PI may be no change in treated mice compared to untreated mice. In this study, we could identify and quantify the levels of tadalafil in the placenta and in the fetal brain. Because tadalafil has been reported to have central effects^20^, other mechanisms through which tadalafil could directly have an influence on the fetal brain cannot be excluded.

Fetal hypoxia secondary to uteroplacental vascular disease carries a risk of neural disorders. In particular, the WM regions and the hippocampus of the developing brain are vulnerable to hypoxia^2^. Wu *et al*. have recently shown WM microstructural alterations in children with ADHD using imaging data^21^. The dorsal hippocampus is associated with the cognitive functions that are impaired in ADHD children^22^. Rodent models of FGR exposed to hypoxia have shown that astrocyte accumulation and impaired myelination accompanies the WM injury after birth^23^. Additionally, the protein level of synaptophysin has been reported to be reduced in the hippocampus of FGR animals on P35 using a utero-placental insufficiency model^24^. The difference between the C and TL groups in synaptophysin levels was greater on P30 than on P15. The neuroinflammation and microglia-mediated pruning may be involved. These findings were confirmed in the FGR offspring of the L-NAME-induced mouse model. Taken together with the results of HIF-2α expression in the fetal brain, prenatal hypoxia conditions play an important role in the impaired neurodevelopment of FGR offspring in our mouse model. Because synaptogenesis peaks during postnatal week two and myelination peaks at approximately P20 in rodents^25^, our results indicated that prenatal hypoxia conditions have an adverse effect on the developing brain during the critical period of both synaptogenesis and myelination. We also showed that tadalafil treatment *in utero* improved synaptogenesis and myelination in offspring on P15 and P30 in this study. Furthermore, astrocyte accumulation in the corpus callosum was reduced by tadalafil treatment. These results suggest that tadalafil treatment for PE with FGR not only ameliorates maternal hypertension and facilitates fetal growth, but also has neuro-benefit effects on the developing brain of FGR offspring. However, synaptogenesis and myelination were suppressed in tadalafil-treated FGR offspring compared to the control offspring on P30. Camprubí *et al*. have recently reported that physical training can improve the neurological outcome of FGR animals^24^. Further studies are needed to find additional treatment options to tadalafil treatment to achieve better neurological outcomes of FGR offspring.

In conclusion, tadalafil treatment for PE with FGR not only ameliorates maternal hypertension and facilitates fetal growth, but also has neuro-benefit effects on the developing brain of FGR offspring through modulation of prenatal hypoxic conditions.

## Methods

The present study was conducted in accordance with the principles and procedures outlined by the Ethics Committee for Animal Research of the Mie University Graduate School of Medicine.

### Study 1: Effect of tadalafil treatment for FGR on the fetal brain

We used the FGR mouse model induced by L-NG-nitroarginine methyl ester (L-NAME) as described previously, with a small modification^13^. Eighteen pregnant C57BL/6 mice (CLEA Japan, Tokyo, Japan) were purchased nine days postcoitum (d.p.c.). Food and water were available *ad libitum*. Dams were matched by weight and divided into two groups 11 d.p.c. A control group of dams (n = 5) received 0.5% carboxymethylcellulose (CMC; Wako Pure Chemical Industries, Osaka, Japan) dissolved in drinking water (C dam). L-NAME-treated groups (n = 13) received 1 mg/ml L-NAME (Cayman Chemical, Ann Arbor, MI, US) dissolved in 0.5% CMC. The L-NAME-treated dams were matched by weight and systolic blood pressure (SBP), and were divided into two subgroups 14 d.p.c. SBP was measured by an indirect tail-cuff method (MK-2000; Muromachi Kikai Co., Tokyo, Japan). One subgroup continued to receive only L-NAME (L dam, n = 8) while the other subgroup received L-NAME with 0.08 mg/ml tadalafil (Cayman Chemical Company, Ann Arbor, MI, US) suspended in 0.5% CMC (TL dam, n = 5). Maternal SBP was again measured 16 d.p.c. by an indirect tail-cuff method (MK-2000; Muromachi Kikai Co., Tokyo, Japan). The dams received an ultrasound examination (see below) and were sacrificed 17 d.p.c. Placenta and fetal brain were collected for further analysis.

We analyzed in no L-NAME group but tadalafil treated (CT dams). CT dams was the same result as C dams. The data of CT dams is not described in this paper.

### Ultrasound biomicroscopy for assessment of blood flow in the fetal brain

Ultrasound biomicroscopy was performed using the anesthetized mouse fixed in a supine position on a heating pad with imbedded electrocardiography leads. The level of anesthesia was adjusted from 0.5% to 1.5% sevoflurane (Pfizer Japan Inc., Tokyo, Japan) to maintain a maternal heart rate from 450 to 550 beats per minute to avoid fetal bradycardia^24^. Maternal temperature was maintained between 36.5 °C to 37.5 °C. Blood flow in the fetal brain was assessed using a high-frequency ultrasound biomicroscope (Vevo2100, VisualSonics, Toronto, Ontario, Canada) with the MS-400 convex probe (24 MHz). The maximum and minimum velocities of the fetal MCA near the circle of Willis were obtained by pulsed wave Doppler images. The mean value of three clear consecutive waveforms was recorded and the average maximum and minimum velocities of the fetal MCA and the fetal MCA PI were calculated in each dam. The angle of insonation was kept permanently below 60°. These parameters were measured in three fetuses randomly taken as representative of the litter. The mean value for each parameter was obtained from the three fetuses in the same litter and was considered as one sample.

### Measurement of the concentration of tadalafil in the placenta and in the fetal brain

Two placentas and two fetal brains were randomly taken as representative of the litter and pooled as one sample. These samples were homogenized by sonication in the presence of 50 mM Tris-HCl (pH 7.3), 100 mM NaCl, 5 mM EDTA, 1 mM EGTA (all from Sigma-Aldrich, St Louis, MO, US), and a protease inhibitor cocktail (Nacalai tesque, Kyoto, Japan). Homogenates were centrifuged at 10000 *g* and 4 °C for 10 min, and the supernatants were collected. Protein content was measured using the Qubit protein assay kit and Qubit 3.0 (Invitrogen, Carlsbad, CA, US)^13^. The concentrations of tadalafil in the placenta and in the fetal brain were confirmed by high-performance liquid chromatography analysis (HPLC)^13^. The limit of detection of tadalafil by HPLC was 3 ng/ml.

### Histological analysis and immunohistochemistry of placenta and fetal brain

One placenta and one fetus were randomly taken as representative of the litter and used for this histological assessment in Study 1. Placenta and fetal brain were fixed in 4% paraformaldehyde (Nacalai tesque, Kyoto, Japan) in 0.2 M sodium phosphate buffer (PBS) (pH 7.4) and then embedded in paraffin (Merck Ltd., Frankfurter, Germany) using standard procedures. The paraffin blocks were cut into 5-μm sections. Coronal brain slices were chosen according to the Electronic Prenatal Mouse Brain Atlas to include the WM regions (Supplementary Fig. S3A), the DG regions of the fetal hippocampus, and the CA regions of the fetal hippocampus (Supplementary Fig. S3B)^26^. Placenta sections were incubated at room temperature overnight with anti-HIF-2α antibody (1:100, Abcam, Cambridge, UK) and fetal brain sections were incubated with anti-HIF-2α antibody (1:4000, Abcam, Cambridge, UK). Sections were incubated with goat anti-rabbit IgG for 3 h, then incubated with peroxidase antiperoxidase complex for 2 h. Sections were then incubated with 3,3′-diaminobenzidine (DAB substrate kit; Vector Laboratories, Burlingame, CA, US).

Sections were scanned using an Olympus IX71 microscope (Olympus, Tokyo, Japan). The average number of HIF-2α+ cells in the labyrinth zone of the placenta was manually counted in four randomly chosen fields (165μm × 220μm) in each section as described previously^25^. We have randomly chosen the field placed anatomical structure of interest to make sure that there are no overlaps. The three counting fields for white matter (WM) regions in the fetal brain, a square of 75 μm × 65 μm, was randomly placed at the anatomical structures of interest. The three counting fields for the hippocampus in the fetal brain was randomly placed corresponding to the DG regions of the fetal hippocampus with a square of 60 μm × 60 μm, and the CA regions of the fetal hippocampus with a square of 50 μm × 50 μm.

### Study 2: Effect of tadalafil treatment for FGR on the developing brain of the offspring

Another set of dams (C dams, n = 4; L dams, n = 5; TL dams, n = 6) was prepared as described above in Study 1, and the dams were allowed to deliver spontaneously. To limit the litter size to 6 to 9 pups per dam, pups were culled and fostered to other dams by 48 h post delivery^27^. All dams were given normal drinking water during lactation.

The pups were anesthetized with sevoflurane (Pfizer Japan Inc., Tokyo, Japan) and perfusion-fixed with 4% paraformaldehyde (Nacalai tesque, Kyoto, Japan) in 0.2 M PBS (pH 7.4) on postnatal day 15 (P15: control offspring (C offspring), n = 5; L-NAME offspring (L offspring), n = 5; L-NAME with tadalafil offspring (TL offspring), n = 4) or P30 (C offspring, n = 4; L offspring, n = 4; TL offspring, n = 3). Their brains were removed for immunohistological analysis. Only male pups were used in this study to minimize the possible influence of sexual dimorphism.

### Fluorescent immunohistochemistry of the brain in offspring

The brains were fixed in 4% paraformaldehyde (Nacalai tesque, Kyoto, Japan) in 0.2 M PBS (pH 7.4) and then embedded in paraffin (Merck Ltd., Frankfurt, Germany) using standard procedures. The paraffin blocks were cut into 5-μm sections. Sections were incubated overnight at room temperature with rabbit anti-GFAP polyclonal antibody (1:300; Abcam, Cambridge, UK), rat anti-MBP monoclonal antibody (1:50; Abcam, Cambridge, UK), or mouse anti-synaptophysin monoclonal antibody (1:100; Millipore, Darmstadt, Germany), followed by an appropriate secondary antibody for 2 h at room temperature.

Fluorescent images were acquired using an FV1000-D IX81 confocal laser microscope (Olympus, Tokyo, Japan). The average number of GFAP positive (GFAP+) cells in the corpus callosum at the striatal level on P15 or P30 was manually counted in five randomly chosen fields (120 μm × 260 μm) in each of three sections per animal, spaced 350 μm apart^28^. The ratios of the areas positively stained with MBP in the cingulum and synaptophysin in the dorsal hippocampus on P15 or P30 were analyzed using Image J software (National Institutes of Health, US) in three sections per animal, spaced 350 μm apart^29–33^.

### Statistical analysis

All values were presented as mean ± SD. All statistical tests were carried out using GraphPad Prism6 (GraphPad Inc.). One-way ANOVA followed by Tukey’s post-hoc test was used for multiple comparisons. *P*-values < 0.05 were considered statistically significant.

### Approvals

All experiments were carried out in accordance with Guidelines for Proper Conduct of Animal Experiments (Science Council of Japan, 2006) for the use of laboratory animals in chronic experiments. Experiments were also approved by the Ethics Committee for Animal Research of the Mie University Graduate School of Medicine(approval number: 29–20).

## Electronic supplementary material

Supplementry Information
