# Supplementary material for: Tadalafil treatment in mice for preeclampsia with fetal growth restriction has neuro-benefic effects in offspring through modulating prenatal hypoxic conditions
Source: Sci Rep. 2019 Jan 18;9:234. doi: 10.1038/s41598-018-36084-x (PMC6338749; doi:10.1038/s41598-018-36084-x)

***Supplementary Information***

**Title: Tadalafil treatment in mice for preeclampsia with fetal growth restriction has neuro-benefic effects in offspring through modulating prenatal hypoxic conditions**

Ryota Tachibana,^1,8^ Takashi Umekawa,^✝^ Kento Yoshikawa,^1^ Takao Owa,^2^ Shoichi Magawa,^1^ Fumi Furuhashi,^1^ Makoto Tsuji,^1^ Shintaro Maki,^1^ Kyoko Shimada,^1^ Michiko K Kaneda,^1^ Masafumi Nii,^1^ Hiroaki Tanaka,^1*^ Kayo Tanaka,^1^ Yuki Kamimoto,^1^ Eiji Kondo,^1^ Ineko Kato,^1^ Kenji Ikemura,^3^ Masahiro Okuda,^3^ Ning Ma,^4^ Takekazu Miyoshi,^5,7^ Hiroshi Hosoda,^6^ Masayuki Endoh,^2^ Tadashi Kimura,^2^ and Tomoaki Ikeda.^1^

^1^Department of Obstetrics and Gynecology, Mie University Graduate School of Medicine, Tsu, Japan.

^2^Department of Obstetrics and Gynecology, Osaka University Graduate School of Medicine, Osaka, Japan.

^3^Department of Pharmacy, Mie University Hospital, Tsu, Japan.

^4^Faculty of Health Science, Suzuka University of Medical Science, Suzuka, Japan ^5^Department of Perinatology and Gynecology, ^6^Department of Regenerative Medicine and Tissue Engineering, National Cerebral and Cardiovascular Center, Suita, Japan

^7^Clinical Research Support Center, Mie University Hospital, Tsu, Japan

^8^These authors equally contributed to this study.

^✝^Deceased

***Corresponding author:** Hiroaki Tanaka, M.D., PhD

2-174 Edobashi, Tsu city, Mie, Japan

Zip code. 514-8507

E-mail. hakumei.s52@gmail.com

Phone. +81-59-232-1111

Fax. +81-59-231-5202

**Supporting Information**

**Table S1. Maternal body weight and food intake during pregnancy in Study 1.**

**Fig. S1. Maternal mean systolic blood pressure (SBP) 14 d.p.c. and 16 d.p.c.**

Maternal mean SBP 14 d.p.c. (A) and 16 d.p.c. (B) for C dam (n = 5), L dam (n = 8),

and TL dam (n = 5). Values are presented as mean ± SD. Asterisks show statistically

significant differences (P < 0.05) between groups indicated by square brackets as determined by one-way ANOVA followed by Tukey’s post-hoc test.

**Fig. S2. Fetal body weight (BW) and placental weight 17 d.p.c.**

Fetal BW 17 d.p.c. for C fetus (n = 40), L fetus (n = 68), and TL fetus (n = 37) (A), and

placental weight 17 d.p.c. for C placenta (n = 40), L placenta (n = 68), and TL placenta

(n = 37) (B). Values are presented as mean ± SD. Asterisks show statistically significant differences (P < 0.05) between groups indicated by square brackets as determined by one-way ANOVA followed by Tukey’s post-hoc test. C fetus and placenta: fetus and placenta from C dam. L fetus and placenta: fetus and placenta from L dam. TL fetus and placenta: fetus and placenta from TL dam.

**Fig. S3. Representative images of the white matter, the dentate gyrus and the　cornu ammonis of the hippocampus in the fetal brain 17 d.p.c.**

(A) White matter. WM: white matter. (B) The dentate gyrus and the cornu ammonis of the hippocampus. DG: dentate gyrus. CA: cornu ammonis. The coronal brain slices

were chosen in cresyl violet-stained sections according to the Electronic Prenatal Mouse Brain Atlas*. Scale bars: 200 μm.

**Reference**

*EPMBA - The Electronic Prenatal Mouse Brain Atlas. Available at:

http://www.epmba.org/. (Accessed: 5th January 2018)

**Supplementary Results**

**Table S1. M** **aternal body weight and food intake during pregnancy in Study 1.**
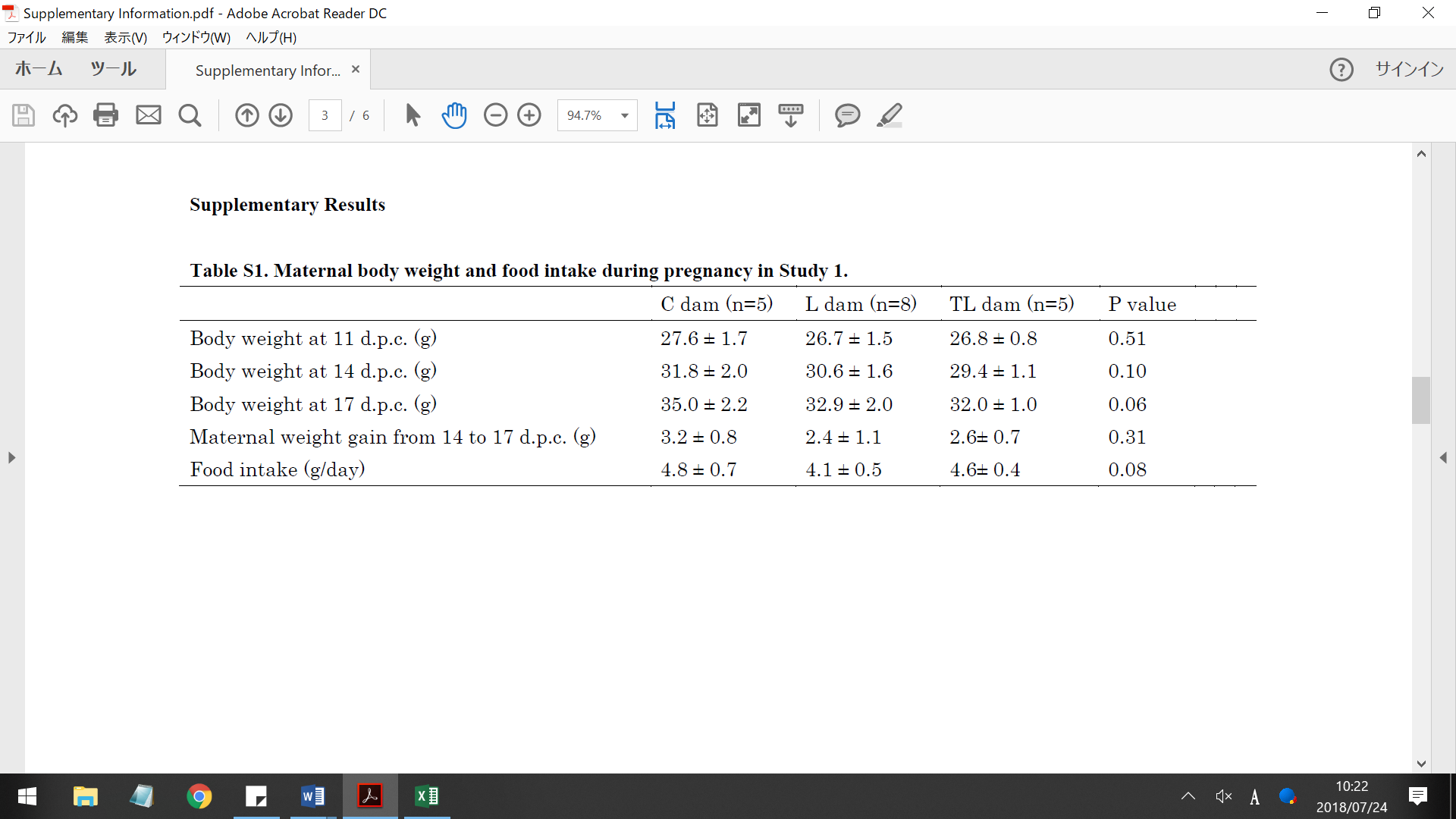


**Supplementary Fig. S1**


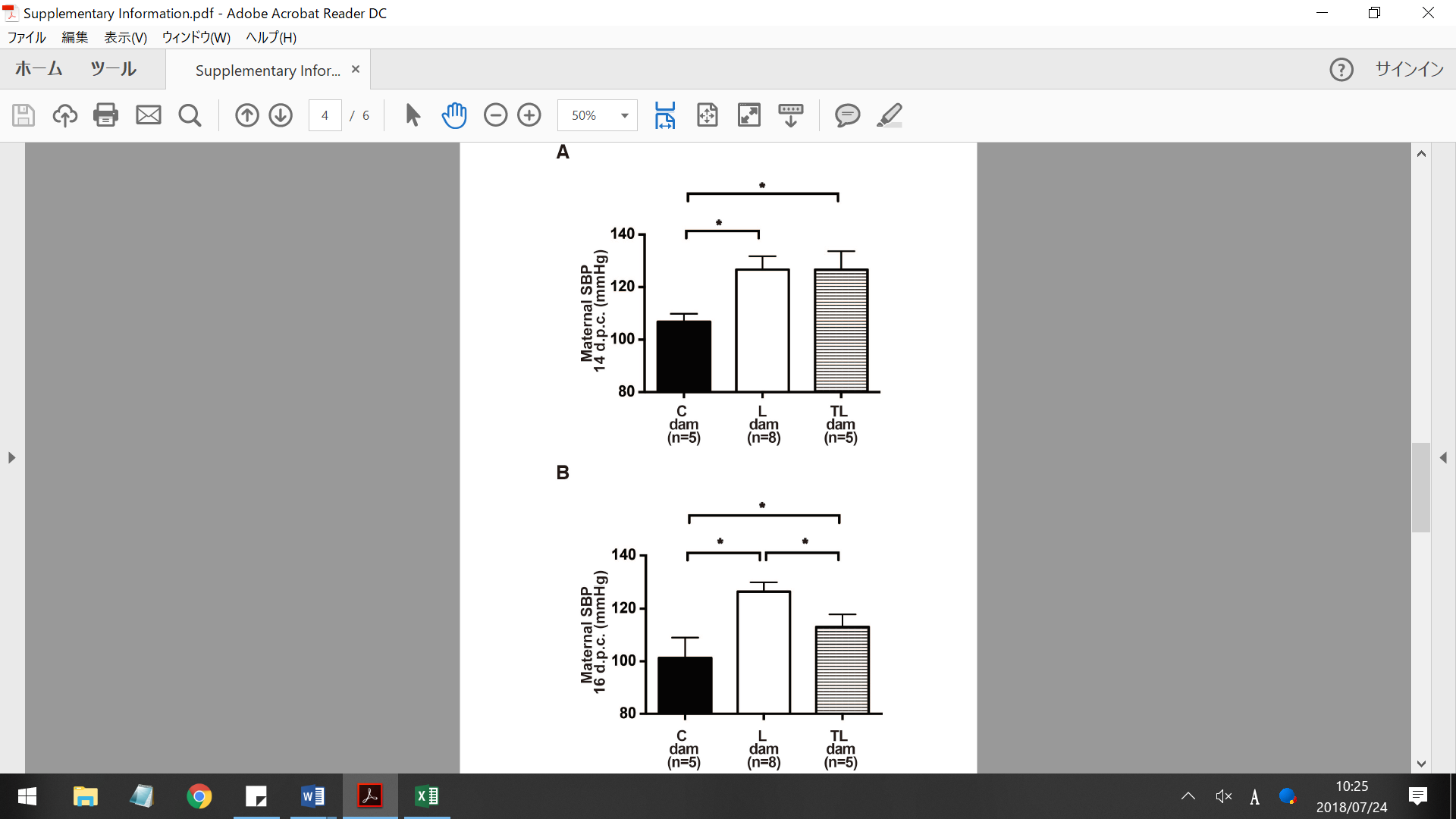


**Supplementary Fig. S2**


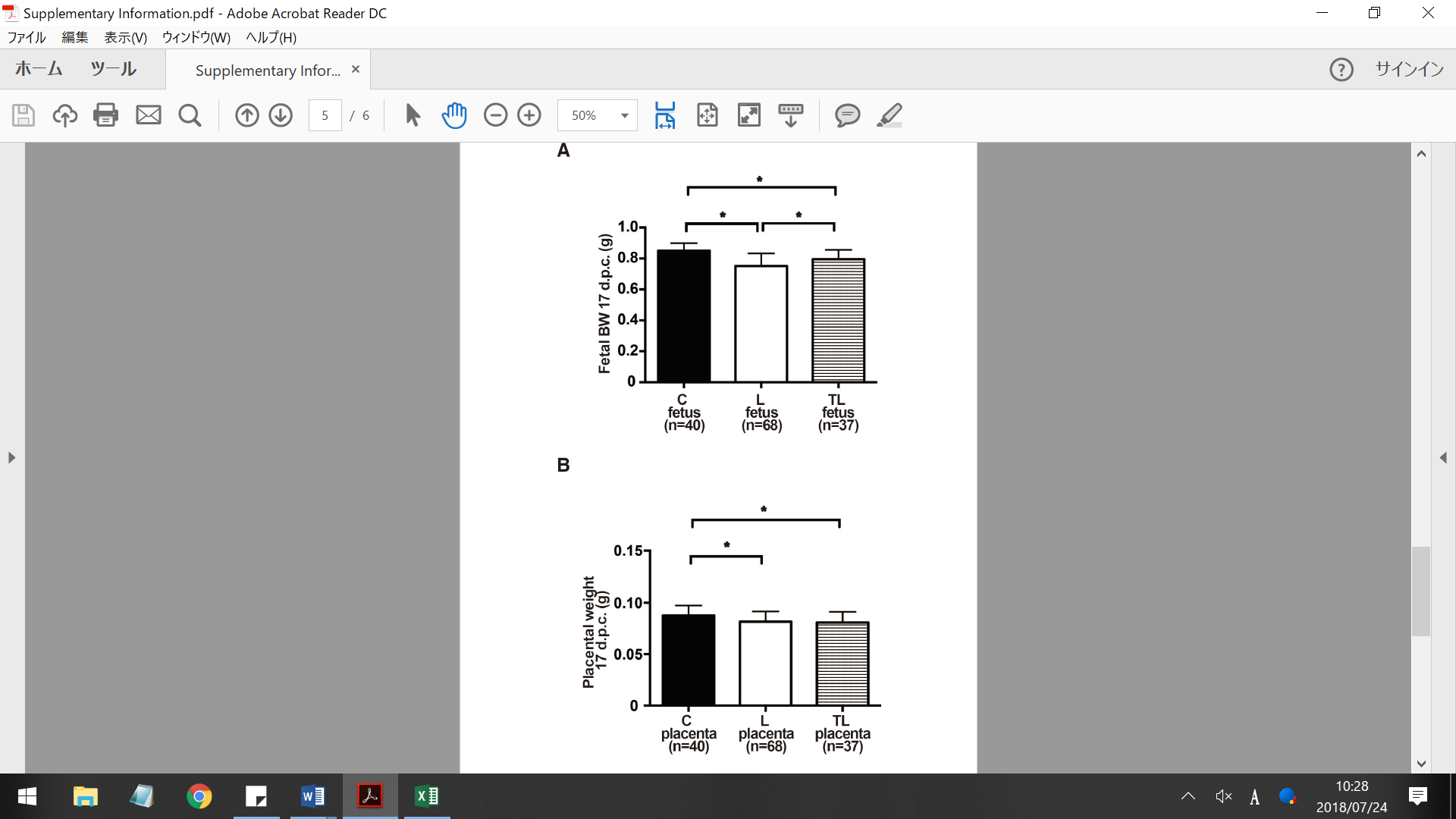


**Supplementary Fig. S3**


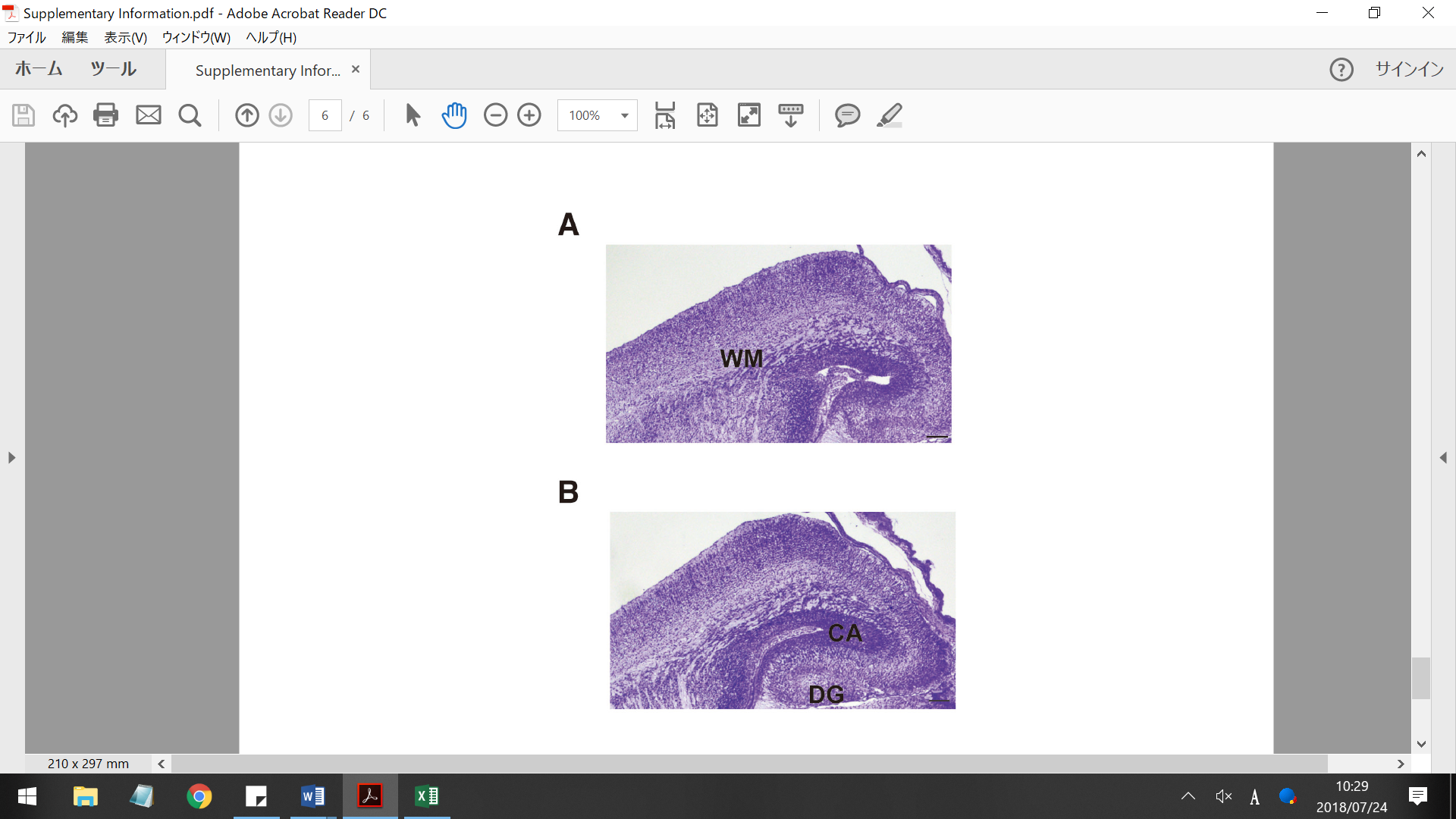

Supplement: Supplementary file 1 — Supplementry Information [file 41598_2018_36084_MOESM1_ESM.docx]
